# Eye Movement Abnormalities in Amyotrophic Lateral Sclerosis

**DOI:** 10.3390/brainsci12040489

**Published:** 2022-04-11

**Authors:** Xintong Guo, Xiaoxuan Liu, Shan Ye, Xiangyi Liu, Xu Yang, Dongsheng Fan

**Affiliations:** 1 Department of Neurology, Peking University Third Hospital, Beijing 100191, China; guoxintong@bjmu.edu.cn (X.G.); luxixi214@sina.com (X.L.); yeshanbysy@163.com (S.Y.); liuxiangyi@bjmu.edu.cn (X.L.); 2 Beijing Municipal Key Laboratory of Biomarker and Translational Research in Neurodegenerative Diseases, Beijing 100191, China; 3 Department of Neurology, Aerospace Center Hospital, Beijing 100049, China; yangxu2011@163.com; 4 Key Laboratory for Neuroscience, Ministry of Education/National Health Commission, Peking University, Beijing 100191, China

**Keywords:** amyotrophic lateral sclerosis, eye movement abnormalities, bulbar involvement

## Abstract

It is generally believed that eye movements are completely spared in amyotrophic lateral sclerosis (ALS). Although a series of eye movement abnormalities has been recognized in recent years, the findings are highly controversial, and bulbar disabilities should be considered in relation to eye movement abnormalities. The present study aimed to determine whether eye movement abnormalities are present in ALS and, if so, to investigate their characteristics and their association with bulbar disability in ALS patients. A total of 60 patients and 30 controls were recruited and underwent the standardized evaluations of the oculomotor system using videonystagmography. Square-wave jerks (OR: 16.20, 95% CI: 3.50–74.95, *p* < 0.001) and abnormal cogwheeling during smooth pursuit (OR: 14.04, 95% CI: 3.00–65.75, *p* = 0.001) were more frequently observed in ALS patients than in the control subjects. In subgroup analyses, square-wave jerks (OR: 26.51, 95% CI: 2.83–248.05, *p* = 0.004) and abnormal cogwheeling during smooth pursuit (OR: 6.56, 95% CI: 1.19–36.16, *p* = 0.031) were found to be more common in ALS patients with bulbar involvement (*n* = 44) than in those without bulbar involvement (*n* = 16). There were no significant differences in the investigated eye movement parameters between bulbar-onset (*n* = 12) and spinal-onset patients (*n* = 48). ALS patients showed a range of eye movement abnormalities, affecting mainly the ocular fixation and smooth pursuit systems. Our pioneering study indicates that the region of involvement could better indicate the pathophysiological essence of the abnormalities than the type of onset pattern in ALS. Eye movement abnormalities may be potential clinical markers for objectively evaluating upper brainstem or supratentorial cerebral lesion neurodegeneration in ALS.

## 1. Introduction

Amyotrophic lateral sclerosis (ALS) is a rare but fatal progressive neurodegenerative disorder involving both upper and lower motor neurons in the cerebral cortex, brainstem nuclei, the anterior horn of the spinal cord, and the corticospinal tract [1,2]. Degeneration of the motor neurons leads to severe weakness and wasting with fasciculations of muscles, disturbed speech and swallowing, and ultimately death due to respiratory failure [3,4,5].

Studies of ALS before the 1980s generally concluded that eye movements were completely spared. Although a series of eye movement abnormalities has been recognized in recent years [6,7,8,9,10,11,12,13,14,15,16,17,18,19,20,21], the findings are highly controversial, and the corresponding pattern has not yet been established. Additionally, previous work from our research group recorded vestibular evoked myogenic potentials (VEMPs) through electromyography in ALS and found that although the clinical examinations of eye movement were normal, alterations were present in ocular VEMPs (o-VEMPs) [22]. This finding also suggested the impairment of the ocular motor system. The pathological 43-kDa transactivating responsive sequence DNA-binding protein (TDP-43) has been identified as the major disease protein in ALS [23]. According to Braak and Brettschneider et al. [24,25], the phosphorylated TDP-43 (pTDP-43) pathology in ALS typically spreads in a sequential pattern along corticofugal axonal pathways, following a four-stage pattern of neuropathological spread. Gorges et al. [26] suggested that eye movement abnormalities are consistent with the progression of pTDP-43 deposition in ALS and occur in a two-stage sequential pattern. Therefore, eye movement abnormalities may provide useful insights into the potential pathophysiological mechanism of the disease.

Given that various types of premotor neurons related to eye movements are located in the brainstem [27], it seems reasonable that bulbar disabilities should be considered in relation to eye movement abnormalities. The objectives of the present study were to determine whether eye movement abnormalities were present in ALS and, if so, to investigate their characteristics and their association with bulbar disability in ALS patients.

## 2. Materials and Methods

### 2.1. Subjects

Sixty patients who fulfilled the Revised El Escorial Criteria [1] for clinically definite, probable, or laboratory-supported probable ALS were recruited to join the study. Additionally, thirty healthy control individuals without any history or clinical manifestations of any neurological disorders and without any familial relations to the gene carriers were included. The exclusion criteria were any other neurodegenerative diseases; any history of eye disorders or vestibular system disease that may affect eye movements; and the use of medications that might influence eye movements, such as diazepam, clonazepam, or other antipsychotics. None of the patients had severe respiratory deficits or were being treated with noninvasive ventilation. All of the subjects were alert and responsive during testing. The study was approved by the institutional ethics committee of Peking University Third Hospital, and all the subjects provided written informed consent prior to inclusion in the study.

### 2.2. Demographic and Clinical Data

Basic demographic and clinical data were collected. Thorough neurological examinations were administered by at least two experienced neurologists for ALS patients. Disease duration was defined as the time from initial symptom onset to the date of oculomotor evaluation. The disease onset pattern was recorded as bulbar onset or spinal onset. According to the presence of upper motor neuron signs or lower motor neuron signs in the bulbar region, the ALS patients were subdivided into two groups: ALS patients with bulbar involvement and ALS patients without bulbar involvement. Upper motor neuron signs included pathologically brisk reflexes, including positive jaw jerks, exaggerated gag reflexes, exaggerated snout reflexes, forced yawning, and pseudobulbar features (mainly uncontrolled crying or laughing) [1,28]. Lower motor neuron signs included atrophy and fasciculations or weakness of bulbar motor neuron-innervated muscles. Additionally, abundant fibrillations and sharp waves could be detected by needle electromyography, and subacute or chronic neurogenic changes, such as giant polyphasic motor unit potentials in the tongue, masseter, and sternocleidomastoid muscle (SCMM), could aid in detecting the presence of subclinical bulbar lower motor neuron involvement. ALS patients were assessed with the Amyotrophic Lateral Sclerosis Functional Rating Scale—Revised (ALSFRS-R) to evaluate disease severity and measure functional limitations [29]. This scale consists of 3 main blocks, including a total of 12 items, for evaluating bulbar function, motor function, and respiratory function; possible scores range from 0 to 48, with lower scores reflecting greater disability. The rate of disability progression (ΔALSFRS-R) was calculated as the drop in ALSFRS-R from a presumed baseline score of 48 divided by the disease duration in months from symptom onset to the oculomotor evaluations [30].

### 2.3. Oculomotor Evaluations

Oculomotor evaluations were performed in a dark room and recorded using videonystagmography (VisualEyes525, Interacoustics, Middelfart, Denmark). All subjects underwent a standardized evaluation by an experienced VNG technologist, and eye movement abnormalities were confirmed by two neurologists who reviewed the oculography findings and video recordings. The distance from the subjects’ eyes to the screen was approximately 120 cm, and the height of the chair was adjusted to ensure that the subjects’ eyes were at approximately at the same height as the target. All subjects were required to keep their heads still while moving their eyes according to the instructions. The equipment was calibrated at the beginning of the test.

Gaze test: The subjects were required to fixate on a yellow light spot (target) in a central position and then in eccentricities of ± 30° horizontally and vertically, 20 s in each position. Saccadic intrusions were classified as involuntary saccades that disrupted fixation. Square-wave jerks, a particular subtype of saccadic intrusion that often occurs in series, were defined as horizontal saccades (amplitude 0.5–15.0°) that moved the gaze away from the target position and then returned it within 200 ms [27]. Gaze-evoked nystagmus (GEN) was defined as nystagmus that increased in intensity as the eyes were brought from the primary gaze position to an eccentric gaze.

Reflexive saccade test: The subjects were asked to follow the pseudorandom target that moved along the horizontal line between ±30° as rapidly and as accurately as possible. Each target step proceeded with the previous step, with intervals of 1.5 s. After the subject completed the reflexive saccade task, an interactive computerized analysis was carried out to quantify the latency and velocity. Saccadic dysmetria included saccade hypermetria and saccade hypometria. The main characteristics of hypermetric and hypometric saccades, respectively, were that the eyes overstepped or lagged behind the target, remained at a fixed point a few degrees beyond or short of the target for approximately 150–200 ms, and then returned to acquire the target [20]. Saccadic dysmetria was diagnosed when the subjects experienced corrective saccades for at least one-third of the total positions during the reflexive saccade test [20].

Smooth pursuit test: Smooth pursuit was studied using a target with sinusoidal movement (amplitude ± 25°) in the horizontal plane for 40 s with the goal of stabilizing a moving image on the fovea. These tests were performed at frequencies of 0.1, 0.2, and 0.4 Hz in increasing order; the subjects were asked to fixate on the target smoothly. Smooth pursuit velocity gain was defined as the relationship between the velocity of the eye and the velocity of the target at a given time and was recorded for the left and right directions. Smooth pursuit velocity gain was calculated separately by computer for the left and right direction. Saccadic pursuit occurred when the eyes lagged behind the target, and the oculomotor system needed to generate saccadic eye movements to correct it. These series of saccadic pursuits were referred to as “cogwheeling” [31].

The presence of square-wave jerks, GEN, saccadic dysmetria, or abnormal cogwheeling during smooth pursuit was labelled in a binary fashion and not further quantified.

### 2.4. Statistical Analysis

Continuous variables were tested for distribution normality using the Shapiro–Wilk test first. Data are reported as the mean ± standard deviation or median (first quartile, third quartile). Categorical variables are expressed as counts and percentages. Independent *t*-tests or Mann–Whitney U tests were applied to continuous variables. The chi-square tests or Fisher’s exact tests were used for categorical variables. Multivariable logistic regression models were finally used to confirm the significant associations between eye movement examinations and clinical features, adjusting for the main potential confounders referred to in previous literature [6,31,32] and those with significant differences in univariate analysis. Odds ratios (ORs) and 95% confidence intervals (CIs) were reported accordingly. All statistical analyses were performed using complex sample survey data in SPSS version 26.0 (SPSS, Chicago, IL, USA). A two-tailed *p* < 0.05 was considered to demonstrate statistical significance.

## 3. Results

### 3.1. Comparison between ALS Patients and Control Subjects

The ALS patients consisted of 30 males and 30 females (mean age 54.35 ± 10.87 years, ranging 28–74 years), and the control subjects consisted of 15 males and 15 females (mean age 53.13 ± 10.50, ranging 29–75 years). There were no significant differences between the groups in age or sex distribution. For the ALS patients, the mean age of onset was 52.82 ± 11.33 years (ranging 28–73 years), and the median duration of disease was 17.50 months (ranging 5–51 months). The median ALSFRS-R total score was 40.00, and the median ΔALSFRS-R was 0.57. A summary of the demographic features and clinical characteristics of ALS patients and control subjects is presented in Table 1.

**Figure 1:**
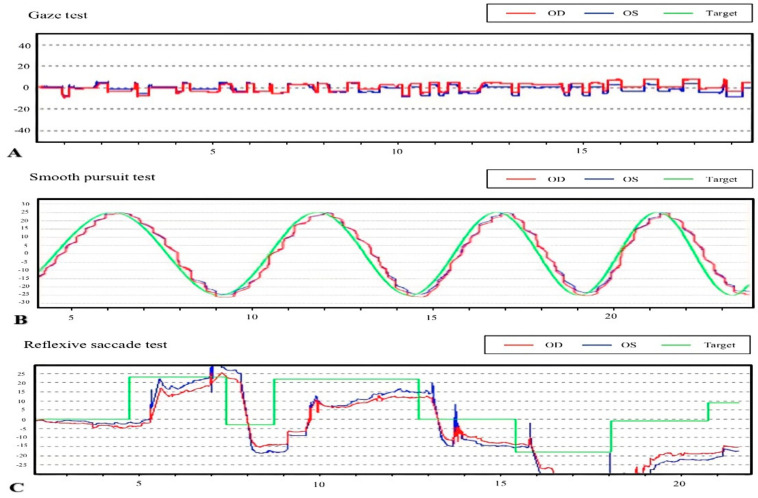
Examples of eye movement abnormalities in our ALS patients. Abbreviations: OD, oculus dexter; OS, oculus sinister. (**A**) Square-wave jerks, (**B**) abnormal cogwheeling during smooth pursuit, and (**C**) saccadic hypometria.

The oculomotor evaluations indicated that eye movement abnormalities, such as square-wave jerks, abnormal cogwheeling during smooth pursuit, and saccade hypometria, were observed in ALS patients (Figure 1). Square-wave jerks were observed in 6.7% (2/30) of the control subjects and in 53.3% (32/60) of the ALS patients, and this difference was statistically significant (adjusted by age; OR: 16.20, 95% CI: 3.50–74.95, *p* < 0.001). A total of 50% (30/60) of the ALS patients and 6.7% (2/30) of the control subjects showed abnormal cogwheeling during smooth pursuit; the difference between the groups was significant (adjusted by age; OR: 14.04, 95% CI: 3.00–65.75, *p* = 0.001). Six (10.0%) ALS patients exhibited saccade hypometria, but no statistically significant differences were noted between ALS patients and controls. Compared with those in control subjects, reflexive saccade latency, reflexive saccade velocity, and smooth pursuit velocity gain were normal in ALS patients. None of the ALS patients or control subjects had either ophthalmoplegia or GEN. Table 2 shows a comparison of the oculomotor performance between ALS patients and control subjects. Table 3 displays the univariate and multivariate logistic regression analyses between clinical data and oculomotor performance of ALS patients and control subjects.

### 3.2. Clinical Data and Oculomotor Performance of ALS Patients with and without Bulbar Involvement

Forty-four ALS patients with bulbar or pseudobulbar signs were classified as ALS patients with bulbar involvement, accounting for 73.3% of all enrolled ALS patients. Compared with ALS patients without bulbar involvement, ALS patients with bulbar involvement had more severe functional limitations, as revealed by the ALSFRS-R total score (*p* = 0.015), and showed a higher ΔALSFRS-R (*p* = 0.009) (Table 4). There were no significant differences in age, sex distribution, age of onset, or disease duration between the groups. Square-wave jerks were detected in 68.2% (30/44) of the ALS patients with bulbar involvement and in 12.5% (2/16) of the ALS patients without bulbar involvement, with a significant difference between the groups (adjusted by age, ALSFRS-R total score and ΔALSFRS-R; OR: 26.51, 95% CI: 2.83–248.05, *p* = 0.004). The percentages of subjects with abnormal cogwheeling during smooth pursuit were 63.6% for the ALS patients with bulbar involvement group and 12.5% for the ALS patients without bulbar involvement group, and the difference between the groups was significant (adjusted by age, ALSFRS-R total score and ΔALSFRS-R; OR: 6.56, 95% CI: 1.19–36.16, *p* = 0.031). Saccade hypometria occurred in one ALS patient without bulbar involvement and five ALS patients with bulbar involvement, but this difference was not statistically significant. Reflexive saccade latency, reflexive saccade velocity, and smooth pursuit velocity gain did not differ significantly between ALS patients with bulbar involvement and ALS patients without bulbar involvement. Table 5 displays the univariate and multivariate logistic regression analyses between clinical data and oculomotor performance of ALS patients with and without bulbar involvement.

### 3.3. Comparison between Bulbar-Onset and Spinal-Onset ALS Patients

There were 12 bulbar-onset and 48 spinal-onset ALS patients. The clinical data and oculomotor performances of bulbar-onset and spinal-onset ALS patients are shown in Table 6. There were no statistically significant differences in demographic or clinical characteristics or the investigated eye movement parameters between the bulbar-onset group and spinal-onset group.

## 4. Discussion

In the present study, eye movement abnormalities, such as square-wave jerks, abnormal cogwheeling during smooth pursuit, and saccade hypometria, were observed in ALS patients. Square-wave jerks and abnormal cogwheeling during smooth pursuit were more frequently observed in ALS patients, especially in ALS patients with bulbar involvement. There were no significant differences in the investigated eye movement parameters between bulbar-onset and spinal-onset patients.

The square-wave jerk rate was markedly higher in ALS patients than in controls. Previous studies have reached a similar conclusion [6,14], but we subsequently conducted subgroup analyses and found that square-wave jerks were more common in ALS patients with bulbar involvement than in ALS patients without bulbar involvement. The genesis of square-wave jerks remains uncertain, and our present findings are supported by the “brainstem hypothesis” of square-wave jerk generation [33]. This theory, based on the model of Otero-Millan et al. [34], assumes the presence of a disturbance in the brainstem neural circuits. These circuits are mainly formed by excitatory burst neurons (EBNs), inhibitory burst neurons (IBNs), omnipause neurons (OPNs), and their connections with the superior colliculus (SC). Furthermore, electrophysiological and pharmacological inactivation studies in monkeys have suggested that the rostral SC is important in sustaining steady fixation [35,36]. When ocular fixation is destabilized—that is, the normal suppression of saccades by the rostral SC is disrupted—OPNs allow EBNs and IBNs to fire and may cause square-wave jerks. ALS patients with bulbar involvement are likely to have more extensive brainstem pathology compared to those without bulbar involvement and are therefore more likely to have damage to the brainstem ocular motor network. With respect to the present findings, we speculate that the increased occurrence rate of square-wave jerks may be due to impairment of the brainstem.

There was no such decline in smooth pursuit velocity gain among the ALS patients and those with bulbar involvement. These findings are similar to those reported by Gizzi et al. [10] and Shaunak et al. [14]; however, several studies have found deceased smooth pursuit velocity gain in ALS patients compared to controls [7,8,12,13,21]. Additionally, we found that abnormal cogwheeling during smooth pursuit was evident more often in ALS patients, especially in ALS patients with bulbar involvement. These abnormal pursuits were also identified by Jacobs et al. [6] and Kang et al. [20]. Smooth pursuit is controlled by cerebro-ponto-cerebellar pathways [27]. The middle temporal (MT)/medial superior temporal (MST) region is important for processing moving stimuli and projects to the frontal eye field (FEF) and supplementary eye field (SEF). These, in turn, project to the nucleus reticularis tegmenti pontis (NRTP), cerebellar dorsal vermis, and fastigial nucleus and are important for smooth pursuit initiation. The MT/MST projects to the dorsolateral pontine nucleus (DLPN), flocculus/paraflocculus, nucleus prepositus hypoglossi, and vestibular nucleus and is important for sustaining smooth pursuit. The present findings provided no evidence of cerebellar abnormalities; Jacobs et al. [6] described one ALS patient with severe bulbar disability who had normal smooth pursuits 1 week before death, and postmortem histopathological examination revealed a loss of neurons and reactive gliosis in the hypoglossal nucleus [6]. This would allow impairments of upper brainstem or supratentorial cerebral lesions, explaining the demonstrated abnormal cogwheeling during smooth pursuit.

Abnormalities of reflexive saccades have been reported, including prolonged saccadic latency [12], reduced saccadic velocity [7,13,37], and saccadic dysmetria [8,20]. However, some studies have also shown no abnormalities of reflexive saccades [10,14]. Our results do not entirely coincide with those of previous studies; the only abnormality recorded in reflexive saccades was saccade hypometria, but no statistically significant differences were found between the ALS patients and controls as well as in subgroup analyses. The neural substrate of reflexive saccades comprises both cortical and subcortical components, with the SC playing a vital role [27]. Neurons in the rostral SC are related to visual fixation, and those in the caudal SC are associated with saccades. This functional differentiation suggests that different neural pathways should exist between the ocular fixation and reflexive saccade systems. Given the heterogeneity of ALS, it is necessary to increase the number of patients to finally determine the pattern of eye movement abnormalities in ALS.

Donaghy et al. [17] and Kang et al. [20] reported that some kinds of eye movement abnormalities were more common in ALS patients with bulbar onset than spinal onset. They attributed these findings to extensive brainstem pathology in bulbar-onset disease. However, no significant differences were observed between the bulbar-onset and spinal-onset groups in the investigated eye movement parameters among our ALS patients. A possible reason for the discrepancy between our results and those observed in the two previous studies could be the fact that when spinal-onset ALS patients undergo oculomotor evaluation, they may also show bulbar disabilities, while the analyses of the previous studies did not describe bulbar impairment among spinal-onset ALS patients. The pathological process underlying ALS is histologically characterized by aggregates of the pathological TDP-43; therefore, the results in the present study suggest that the region of involvement could better indicate the pathophysiological essence of eye movement abnormalities than the type of onset pattern in ALS and provide a more rational explanation for the neural pathways involved and intrinsic pathophysiological mechanism of the disease. With increased understanding of sensitive molecular pathological markers, future neuropathological studies have the potential to identify the neural substrate of these eye movement findings in ALS.

Furthermore, some diseases with clinical manifestations similar to ALS, such as cervical spondylotic amyotrophy (CSA) and multifocal motor neuropathy (MMN), have not been reported to cause eye movement abnormalities. Therefore, oculomotor evaluations might have particular value in helping identify patients who were misdiagnosed.

In addition, this study has some limitations that deserve mention. Our videonystagmography equipment cannot provide the frequency or amplitude of the square-wave jerks. The lack of quantitative characterization likely introduced variability into the data, thus decreasing their statistical power. For further development, automatically supporting diagnosis based on the subject’s eye movements may be a worthwhile application. The associations between these eye movement abnormalities and the severities of functional limitation in ALS patients are not clear and need to be verified through further longitudinal studies. Significantly, eye movement patterns for some neurological conditions (e.g., schizophrenia) have been known to differ both for artificial and naturalistic stimuli. Thus, the usage of artificial stimuli, which is perfectly suitable for an initial assessment of population differences or for using in clinical context, does not necessarily reflect the effect of the eye movement abnormalities on the patients’ lives.

## 5. Conclusions

ALS patients showed a range of eye movement abnormalities affecting mainly the ocular fixation and smooth pursuit systems. These abnormalities were observed more often in ALS patients with bulbar involvement. Our pioneering study suggests that the region of involvement could better indicate the pathophysiological essence of abnormalities than the type of onset pattern in ALS and may provide a more rational explanation for the neural pathways involved and intrinsic pathophysiological mechanism of the disease. Eye movement abnormalities may be potential clinical markers for objectively evaluating upper brainstem or supratentorial cerebral lesion neurodegeneration in ALS.

## Figures and Tables

**Table 1:** Demographic and clinical characteristics of ALS patients and control subjects.

	ALS Patients (*n* = 60)	Control Subjects (*n* = 30)	*p*-Value
Age, y	54.35 ± 10.87	53.13 ± 10.50	0.506
Sex, male/female	30 (50.0%)/30 (50.0%)	15 (50.0%)/15 (50.0%)	
Age of onset, y	52.82 ± 11.33		
Disease duration, mo	17.50 (10.00, 33.75)		
Onset pattern, bulbar/spinal	12 (20.0%)/48 (80.0%)		
Bulbar involvement, yes/no	44 (73.3%)/16 (26.7%)		
ALSFRS-R total score	40.00 (36.00, 43.25)		
ΔALSFRS-R	0.57 (0.22, 0.88)		

Data are presented as the mean ± standard deviation, median (first quartile, third quartile), or *N* (%). Abbreviations: ALS, amyotrophic lateral sclerosis; ALSFRS-R, Amyotrophic Lateral Sclerosis Functional Rating Scale—Revised.

**Table 2:** Comparison of oculomotor performance between ALS patients and control subjects.

		ALS Patients (*n* = 60)	Control Subjects (*n* = 30)	*p*-Value
Gaze test	Square-wave jerks	32 (53.3%)	2 (6.7%)	<0.001 *
Reflexive saccade test	Hypometria	6 (10.0%)	0	0.173
	Latency, ms	342.23 ± 47.54	340.79 ± 23.16	0.153
	Velocity, °/s	603.00 (548.00, 656.50)	541.00 (477.50, 657.50) a	0.187
Smooth pursuit test	Abnormal cogwheeling	30 (50.0%)	2 (6.7%)	<0.001 *
	Velocity gain toward left	0.85 (0.82,0.88)	0.87 (0.85,0.89) a	0.106
	Velocity gain toward right	0.85 (0.82,0.87)	0.86 (0.84,0.87) a	0.097

Data are presented as the mean ± standard deviation, median (first quartile, third quartile), *N* or *N* (%). Abbreviations: ALS, amyotrophic lateral sclerosis. a, Normally distributed data but summarized here as the median (first quartile, third quartile). * *p* < 0.05.

**Table 3:** Univariate and multivariate logistic regression analyses between clinical data and oculomotor performance of ALS patients and control subject.

	Square-Wave Jerks				Abnormal Cogwheeling during Smooth Pursuit			
	Univariate Analysis		Multivariable Analysis		Univariate Analysis		Multivariable Analysis	
	OR (95% CI)	*p*-Value	OR (95% CI)	*p*-Value	OR (95% CI)	*p*-Value	OR (95% CI)	*p*-Value
ALS patient	16.00 (3.49–73.27)	<0.001 *	16.20 (3.50–74.95)	<0.001 *	13.50 (2.94–61.90)	0.001 *	14.04 (3.00–65.75)	0.001 *
Age	1.03 (0.99–1.08)	0.135	1.03 (0.99–1.08)	0.160	1.04 (1.00–1.09)	0.050	1.05 (1.00–1.10)	0.056
Sex	0.68 (0.29–1.61)	0.385			0.44 (0.18–1.06)	0.067		

Abbreviations: ALS, amyotrophic lateral sclerosis; ALSFRS-R, Amyotrophic Lateral Sclerosis Functional Rating Scale—Revised. * *p* < 0.05.

**Table 4:** Clinical data and oculomotor performance of ALS patients with and without bulbar involvement.

		ALS Patients with Bulbar Involvement (*n* = 44)	ALS Patients without Bulbar Involvement (*n* = 16)	*p*-Value
Age, y		55.16 ± 11.10	52.13 ± 10.22	0.343
Sex, male/female		22 (50.0%)/22 (50.0%)	8 (50.0%)/8 (50.0%)	
Age of onset, y		53.80 ± 11.66	50.13 ± 10.24	0.271
Disease duration, mo		16.00 (9.25, 32.00)	23.50 (11.25, 41.50)	0.123
ALSFRS-R total score		38.00 (32.00, 41.00)	42.00 (40.00, 44.25)	0.015 *
ΔALSFRS-R		0.71 (0.33, 1.00) a	0.26 (0.15, 0.49)	0.009 *
Gaze test	Square-wave jerks	30 (68.2%)	2 (12.5%)	<0.001 *
Reflexive saccade test	Hypometria	5 (11.4%)	1 (6.3%)	
	Latency, ms	340.64 ± 50.85	346.67 ± 37.95	0.677
	Velocity, °/s	582.00 (548.50, 651.50)	613.00 (517.00, 679.00) a	0.443
Smooth pursuit test	Abnormal cogwheeling	28 (63.6%)	2 (12.5%)	0.001 *
	Velocity gain toward left	0.85 (0.82, 0.88)	0.86 (0.82, 0.90) a	0.437
	Velocity gain toward right	0.85 (0.82, 0.87)	0.85 (0.82, 0.86) a	0.760

Data are presented as the mean ± standard deviation, median (first quartile, third quartile), or *N* (%). Abbreviations: ALS, amyotrophic lateral sclerosis; ALSFRS-R, Amyotrophic Lateral Sclerosis Functional Rating Scale—Revised. a, Normally distributed data but summarized here as the median (first quartile, third quartile). * *p* < 0.05.

**Table 5:** Univariate and multivariate logistic regression analyses between clinical data and oculomotor performance of ALS patients with and without bulbar involvement.

	Square-Wave Jerks				Abnormal Cogwheeling during Smooth Pursuit			
	Univariate Analysis		Multivariable Analysis		Univariate Analysis		Multivariable Analysis	
	OR (95% CI)	*p*-Value	OR (95% CI)	*p*-Value	OR (95% CI)	*p*-Value	OR (95% CI)	*p*-Value
Bulbar involvement	15.00 (2.99–75.17)	0.001 *	26.51 (2.83–248.05)	0.004 *	12.25 (2.46–60.91)	0.002 *	6.56 (1.19–36.16)	0.031 *
Age	1.03 (0.98–1.08)	0.319	1.20 (0.95–1.09)	0.564	1.04 (0.99–1.09)	0.123	1.01 (0.95–1.07)	0.705
Sex	2.26 (0.80–6.36)	0.123			2.36 (0.72–8.70)	0.143		
Age of onset	1.03 (0.98–1.07)	0.294			1.04 (0.99–1.09)	0.126		
Disease duration,	0.98 (0.94–1.02)	0.316			0.98 (0.95–1.02)	0.399		
ALSFRS-R total score	0.92 (0.83–1.02)	0.126	0.97 (0.84–1.12)	0.668	0.90 (0.81–1.01)	0.063	0.96 (0.84–1.09)	0.507
ΔALSFRS-R	2.53 (0.68–9.42)	0.166	0.745 (0.12–4.84)	0.758	3.68 (0.93–14.49)	0.063	1.47 (0.25–8.73)	0.669

Abbreviations: ALS, amyotrophic lateral sclerosis; ALSFRS-R, Amyotrophic Lateral Sclerosis Functional Rating Scale—Revised. * *p* < 0.05.

**Table 6:** Clinical data and oculomotor performance of bulbar-onset and spinal-onset ALS patients.

		Bulbar Onset (*n* = 12)	Spinal Onset (*n* = 48)	*p*-Value
Age, y		58.17 ± 11.58	53.40 ± 10.60	0.176
Sex, male/female		8 (66.7%)/4 (33.3%)	22 (45.8%)/26 (54.2%)	0.197
Age of onset, y		61.50 (45.00, 66.25)	51.00 (44.25, 60.50) a	0.109
Disease duration, mo		11.50 (9.00, 30.00)	20.00 (11.00, 33.75)	0.242
ALSFRS-R total score		40.00 (38.00, 44.50)	40.00 (35.50, 42.50)	0.358
ΔALSFRS-R		0.33 (0.23, 0.86)	0.58 (0.21, 0.88) a	0.746
Gaze test	Square-wave jerks	9 (75.0%)	23 (47.9%)	0.115
Reflexive saccade test	Hypometria	1 (8.3%)	5 (10.4%)	
	Latency, ms	322.17 ± 37.18	347.58 ± 48.91	0.100
	Velocity, °/s	554.00 ± 133.28	587.84 ± 108.60	0.365
Smooth pursuit test	Abnormal cogwheeling	8 (66.7%)	22 (45.8%)	0.333
	Velocity gain toward left	0.85 (0.83, 0.87) a	0.85 (0.82, 0.88)	0.844
	Velocity gain toward right	0.84 (0.82, 0.87) a	0.85 (0.82, 0.86)	0.783

Data are presented as the mean ± standard deviation, median (first quartile, third quartile), or *N* (%). Abbreviations: ALS, amyotrophic lateral sclerosis; ALSFRS-R, Amyotrophic Lateral Sclerosis Functional Rating Scale—Revised. a, Normally distributed data but summarized here as the median (first quartile, third quartile).

## Data Availability

The data presented in this study are available upon request from the corresponding author. The data are not publicly available due to data management regulations in our hospital.
